# Protocol for a randomized controlled trial to test the acceptability and adherence to 6-months of walnut supplementation in Chinese adults at high risk of cardiovascular disease

**DOI:** 10.1186/s12937-020-00660-7

**Published:** 2021-01-06

**Authors:** Yishu Liu, Nan Li, Ni Yan, Xiong-fei Pan, Qiang Li, Renata Micha, Dariush Mozaffarian, Mark D. Huffman, Yanfang Wang, Bruce Neal, Maoyi Tian, Yi Zhao, Jason H. Y. Wu

**Affiliations:** 1 The George Institute for Global Health, Faculty of Medicine, University of New South Wales, Sydney, Australia; 2 The George Institute for Global Health at Peking University Health Science Center, Beijing, China; 3 Ningxia Medical University, Yinchuan, China; 4 Friedman School of Nutrition Science and Policy, Tufts University, Boston, MA USA; 5 Department of Preventive Medicine, Feinberg School of Medicine, Northwestern University, Chicago, IL USA; 6 Peking University Clinical Research Institute, Beijing, China; 7 School of Public Health, Imperial College London, London, UK; 8 Sydney School of Public Health, University of Sydney, Sydney, Australia

**Keywords:** Nuts, Cardiovascular disease, Randomized controlled trial, Plasma ALA

## Abstract

**Background:**

Consumption of nuts improves cardio-metabolic risk factors in clinical trials and relates to lower risk of cardiovascular disease (CVD) in prospective observational studies. However, there has not been an adequately powered randomized controlled trial to test if nuts supplementation actually reduces incident CVD. In order to establish the feasibility of such a trial, the current study aimed to assess the acceptability and adherence to long-term nut supplementation amongst individuals at high CVD risk in China.

**Methods:**

This protocol described a 6-month trial performed in Ningxia Province in China among participants with a history of CVD or older age (female ≥65 years, male ≥60 years) with multiple CVD risk factors. Participants were randomized to control (received non-edible gift), low dose walnut (30 g/d), or high dose walnut (60 g/d) groups in a 1:1:1 ratio. Walnuts were provided at no cost to participants and could be consumed according to personal preferences. Follow-up visits were scheduled at 2 weeks, 3 months and 6 months. The primary outcome was fasting plasma alpha linolenic acid (ALA) levels used as an indicator of walnut consumption. Secondary outcomes included self-reported walnut intake from the 24 h dietary recalls. The target sample size of 210 provided 90% statistical power with two-sided alpha of 0.05 to detect a mean difference of 0.12% (as percent of total fatty acid) in plasma ALA between randomized groups.

**Results:**

Two hundred and ten participants were recruited and randomized during October 2019. Mean age of participants was 65 years (SD = 7.3), 47% were females, and 94% had a history of CVD at baseline. Across the three study groups, participants had similar baseline demographic and clinical characteristics.

**Discussion:**

This trial will quantify acceptability and adherence to long-term walnut supplementation in a Chinese population at high risk of CVD. The findings will support the design of a future large trial to test the effect of walnut supplementation for CVD prevention.

**Trial registration:**

NCT04037943

**Protocol version:** v3.0 August 14 2019

**Supplementary Information:**

The online version contains supplementary material available at 10.1186/s12937-020-00660-7.

## Background

Tree nuts (referred to hereafter as nuts) such as walnuts, almonds, and pistachios are rich in unsaturated fats, soluble fibre, phytosterols, and antioxidants. In meta-analyses of short-term randomized controlled trials (RCT), increased intake of nuts improves cardiovascular disease (CVD) risk factors such as total cholesterol, LDL cholesterol, apolipoprotein B, and triglycerides, as well as markers of endothelial function and glycemic control [1–3]. Unsaturated fats – about 50% of total fats in most nuts – also significantly improve glycemic control in RCTs [4, 5]. These effects appear dependent on the amount (dose) rather than the types of nuts, although most studies to date have focused on walnuts and almonds [1]. Prospective observational studies also support a beneficial role of nuts for the prevention of CVD. A recent meta-analysis has found that higher nuts intake is associated with lower risk of incident CVD, with about 20% lower risk per daily 1-oz (28 g) serving (RR: 0.79, 95% CI: 0.70, 0.88) [6].

While the findings from short-term trials and prospective cohorts suggest likely metabolic benefits and protection against CVD, and a growing number of dietary guidelines around the world recommend the incorporation of nuts as part of a healthy dietary pattern [7–9], several key questions remain unresolved. First, no long-term RCTs have tested the effects of nuts consumption on the risk of CVD. The PREDIMED study, a RCT among Spanish adults, has found that advice to consume a Mediterranean diet supplemented with mixed nuts (30 g/day) significantly reduced incident CVD, compared to advice to follow a low-fat diet [10]. However, the design of PREDIMED does not allow separation of the effects of nuts alone, vs. the Mediterranean dietary advice as a whole. In addition, errors and inconsistencies in the randomization process of PREDIMED, have raised controversy over its findings [11]. Crucially, nearly all the prior trials and cohort studies, including the PREDIMED trial, have been performed in high-income Western populations. The effects of increased nuts intake on CVD in lower- and middle-income countries remain uncertain. For instance, only three controlled trials have been conducted in Chinese participants (2 in China, 1 in Taiwan) [12–14], with several additional trials in other Asian populations [15, 16]. Their findings support likely benefits of nuts supplementation in reducing LDL cholesterol and improving other CVD risk factors. However, all of the trials were short-term (less than 3 months) and not able to test for clinical CVD events.

Cardiovascular disease is the leading cause of death and disability in China [17]. Large dietary surveys indicate that low levels of nuts consumption could be a significant contributor to CVD burden in China, with estimated mean nuts intake of only 5.4 g/day across diverse geographical regions [18]. An appropriately designed and powered RCT to test the effect of nuts supplementation on the risk of incident CVD events in China is therefore of significant scientific and public health importance. We hereby report the protocol for a 6-month walnut supplementation trial in a rural area of China with high rates of CVD, to obtain data on feasibility of long-term walnut supplementation to support and help to refine the design of a future long-term large RCT testing the effect of nuts supplementation on CVD risk. The main objectives of the current trial are to assess the acceptability and adherence to two different doses of walnut supplementation (30 and 60 g per day). The exploratory objectives are to determine the effects of walnut supplementation on cardiovascular risk factors including blood lipids, fasting glucose, and body weight.

## Methods

### Study design

The ‘Nuts for the prevention of cardiovascular disease in Chinese adults’ (NUTS) study was a randomized, controlled, single blind (blinded investigators and blinded assessments of all study endpoints), three-group parallel design trial lasting for 6 months.

**Fig. 1:**
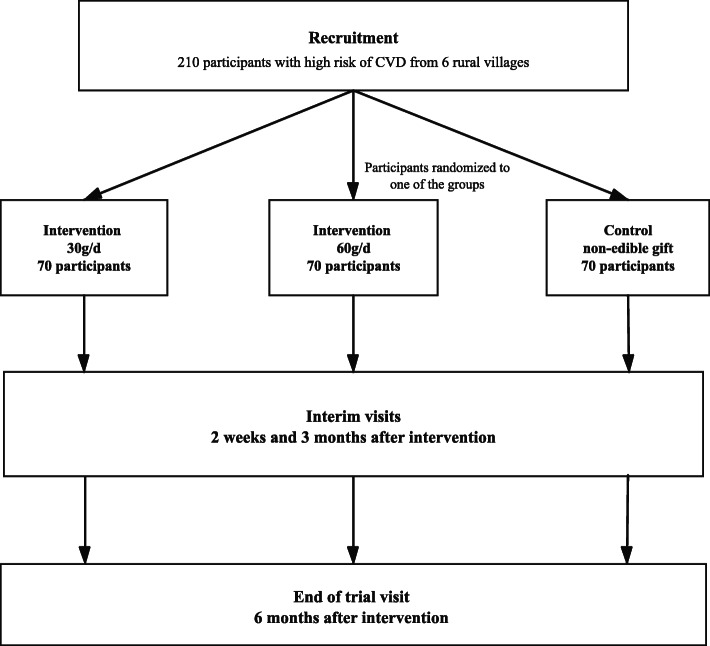
Trial Flowchart. 210 participants with high risk of CVD were recruited from 6 rural villages in Ningxia Province, China. They were randomized in a 1:1:1 ratio to either the control group, or low-dose walnut (30 g/day) intervention group, or high-dose walnut (60 g/day) intervention group; and followed-up for 6 months. Outcome data were collected from participants at study baseline, the interim visits, and at the end of trial visit

Participants were randomized to one of the three groups in a 1:1:1 ratio: control (non-edible gift), low dose (30 g/day) walnut intervention group, and high dose (60 g/day) walnut intervention group (Fig. 1). Written informed consent was obtained from each study participant.

### Study population

We have chosen Ningxia to conduct this trial, since it is a province with high prevalence and mortality rate of stroke and ischemic heart disease [19–21] and the project team has established collaborations with local academic and government institutions. Following consultation with these partners, we recruited adults from six rural villages near Qingtongxia City in Ningxia, based on their willingness to participate and their geographic accessibility. Suitable villages needed to have at least one village doctor who was able to facilitate primary screening of participants.

In each village, we recruited individuals at high risk of CVD based upon having a history of CVD or a high risk of CVD. Inclusion criteria were a history of CVD (defined by prior hospitalization for a myocardial infarction or stroke, unstable angina, coronary artery bypass graft surgery, percutaneous coronary intervention, peripheral revascularization, symptomatic with documented hemodynamically-significant carotid or peripheral vascular disease, or amputation secondary to vascular disease); or older age (≥60 for males, ≥65 years for females), plus at least two of the following CVD risk factors: 1) type 2 diabetes including treatment with at least two oral anti-hyperglycaemic agents, or insulin, 2) systolic blood pressure ≥ 140 mmHg while on one or more antihypertensive agents, 3) current daily smoking, 4) dyslipidaemia defined as HDL-cholesterol < 1.0 mmol/L or LDL-cholesterol > 6.0 mmol/L, or 5) micro- or macro-albuminuria. The definition of high risk individuals was selected to ensure adequate numbers of events and statistical power in the future planned large-scale trial to test walnut supplementation to reduce CVD events. Data from completed and ongoing large-scale trials indicated that patients meeting these criteria experienced an annual event rate of 3.5% for the occurrence of major adverse cardiovascular events [22, 23].

Screening of CVD risk factors was based on self-report with support of physical evidence. Participants were deemed to have the relevant risk factors if they were able to present evidence from a laboratory measurement, prescription drug packaging, tobacco packaging, or clinical report within the past 12 months.

Individuals were excluded if they had never or rarely consumed any types of nuts, had self-reported allergy to nuts (i.e. history of food allergy with hypersensitivity to any of the components of nuts), had other serious medical conditions that prevent nuts consumption (e.g. digestive disease with fat intolerance), had any medical condition thought to limit survival to less than 1 year, had difficulty with consuming nuts (e.g. dental health issues that prevent chewing walnuts), or were unwilling to consume nuts.

### Recruitment and consent

Village doctors distributed study information sheets in their villages and registered individuals who were interested to participate 2 weeks before study recruitment. Potential participants were invited by the village doctors to attend the screening interview at the village clinics. At the screening interview, potential participants were interviewed by trained study staff who explained the background, objectives, and procedures of the trial, sought and obtained written informed consent, confirmed eligibility, and completed the baseline survey. Participants who provided informed consent were invited to complete the baseline fasting blood sample collection the next day. Only one eligible participant was recruited from each household to avoid potential contamination across randomized groups.

### Randomization

Randomization was done centrally by an independent biostatistician using a computer-generated random number sequence after the completion of the baseline survey. Participants were randomized with stratification by village in a 1:1:1 allocation ratio to either one of the intervention groups or to the control group.

### Rationale for the chosen walnut intervention

Walnuts contain a high amount of protein, dietary fibre, vitamins, minerals and phytosterols [24]. Walnuts are a rich source of both omega-6 and omega-3 polyunsaturated fatty acids (linoleic and alpha linolenic acid [ALA]) [25] and improve cardio-metabolic risk factors in short-term clinical feeding trials including lowering LDL-cholesterol [26, 27]. Walnuts have made up the largest proportion (50%) of the mixed nuts used in the PREDIMED study [10]. In addition, walnuts are relatively soft compared to other nut types, making them easier to be chewed and consumed by those with oral health issues such as older adults, who are likely to make up a significant proportion of those recruited to the trial. Walnuts are also readily accessible since China grows a large volume of walnuts [28]. Commercially-available roasted, unsalted walnuts were used in the trial for enhanced taste and longer shelf life. The study walnuts contained 4.47 g of protein, 17.64 g of total fat, 5.73 g of carbohydrate and total energy of 811KJ per 30 g.

A dose-response relationship between greater nuts intake and lower cardio-metabolic risk has been generally observed across a range of nuts intake between 5 g to 100 g per day, with stronger effects for trials providing ≥60 g/day [1, 2]. Since the effect of walnut intake on CVD risk is likely mediated by changes in cardio-metabolic risk factors, we seek to evaluate if a higher dose of daily walnut supplementation is as acceptable as a lower dose. Therefore, for this trial, we tested both 30 g per day (corresponding to many current dietary recommendations), as well as a higher dose of 60 g per day of walnut supplementation.

### Intervention and control

Participants in the intervention groups were provided sachets containing 30 g of roasted and unsalted walnuts free of charge. For the 30 g/day arm, 1 sachet/day was provided, and for the 60 g/day arm, 2 sachets/day were provided. The walnuts were purchased from a local food manufacturer who sourced walnuts from Chinese walnut growers and packaged them in accordance with Chinese food standards (GB/T 20398–2006). The participants were instructed to open and consume the allocated dose of walnuts at any time of the day according to their personal preference, but not to share the walnuts with family members. To ensure freshness, participants were provided with sufficient sachets for the first 3 months of the intervention at baseline and were provided with the remainder at the 3-month follow-up study visit.

Participants in the control group continued with their usual diet without introduction of any nuts supplement. They were provided with small, inedible toiletry gifts at baseline and were provided with similar gifts at each follow-up visit to ensure equal study contacts across all the study groups and encourage attendance at the follow-up visits.

### Data collection and follow up

Informed consent was obtained from each participant before any data collection (Table 1). At baseline, eligible participants completed an interviewer-administered questionnaire, a validated 95-item food frequency questionnaire (FFQ) that asked participants about their usual dietary pattern over the past 12 months, and a 24-h dietary recall [29]. Each participant also underwent physical examination to record height, weight, waist and hip circumferences (Inbody-370S scale and measuring tapes, and anthropometry assessment conducted according to the International Society for the Advancement of Kinanthropometry (ISAK) protocols), and blood pressure (OMRON-7124). After an overnight fast, participants had their blood samples collected at the local township hospital by trained nurses. One blood sample was drawn into 5 ml EDTA-anticoagulated vacutainer tube, and used for measurement of blood lipids (total triglycerides, total cholesterol, LDL-C, and HDL-C) at the township hospital laboratory immediately following routine procedures for the results to be provided to the participants. Another blood samples were drawn into 5 ml EDTA-anticoagulated vacutainer tubes and immediately separated, aliquoted, and stored at − 30 °C in freezers at the township hospital before being couriered (at − 30 °C) to the Ningxia Medical University central laboratory within 72 h of blood collection. All plasma samples were subsequently stored at − 80 °C in the central laboratory. Blood samples will be tested for plasma alpha-linolenic acid (ALA), glucose, LDL-cholesterol, HDL-cholesterol, total cholesterol, and total triglyceride levels. All the specimens will be disposed of 12 months after the end of trial.

**Table 1:** Timeline for key events and collection of outcome data for the NUTS trial

			Follow up 24 weeks				
			Baseline				
Week	−2	−1	0	1	2	12	25^a^
Months			0			3	6
*Pre-intervention screening*							
Information sheets distribution	╳						
Consent for pre-screening	╳	╳					
Pre-screening	╳	╳					
*Baseline*							
Informed consent			╳				
Assessed for eligibility			╳				
Demographics			╳				
Disease history			╳				
Medication use			╳				
Food Frequency Questionnaire			╳				
*Intervention*							
Randomization				╳			
Dispense walnuts (intervention groups)				╳		╳	
Dispense non-edible gifts (control group)				╳	╳	╳	╳
*Endpoint collection*							
Blood pressure			╳				╳
Fasting blood			╳				╳
Anthropometric measurements^b^			╳				╳
24-h dietary recall			╳				╳
Adherence and acceptability (intervention groups)					╳	╳	╳

^a^ 25 week or early withdrawal; ^b^ Weight, height, waist and hip circumference measurements

Interim visits occurred at 2 weeks and 3 months after the baseline for each participant. Blood test results describing blood lipids were provided to participants at the 2-week study visit, with referral to local physicians for management if blood lipid levels exceeded clinical guidelines. Participants in the intervention groups were reminded to consume walnuts every day and completed a 7-item follow-up questionnaire (Supplementary material) related to acceptability and adherence to the walnut supplementation at both interim visits. Participants in the control group did not answer this questionnaire, but received non-edible gifts at each interim visit. At the end of trial (6 months after randomization), all participants completed the same 24-h dietary recall as they did at baseline to assess the change in nuts intake, and fasting blood samples were collected according to the same procedure as at baseline. At the end of the trial, participants from intervention groups again answered the 7-item questionnaire regarding the acceptability and adherence to walnut supplement, and the control group received a non-edible gift (Table 1). All the participants will be notified of the study results once the trial has been completed.

Any condition which required hospitalization was collected at each follow-up visit as adverse event. All adverse events were listed and reported to the ethics committees periodically. Participants who developed food allergy with hypersensitivity to any of the components of walnut in the intervention groups were referred to and had the allergy confirmed by a doctor. Intervention was discontinued among those participants but they were followed up until the end of the trial.

Study personnels were divided into an intervention delivery group (who were not blinded to the allocation of treatment) and an outcome assessment group (who were blinded to the allocation of treatment). The intervention delivery group delivered the walnuts and non-edible gifts and performed the interim study visit questionnaires related to acceptability and adherence. The outcome assessors administered the FFQ, anthropometric assessments, and 24-h dietary recall and collected blood samples from participants at baseline and at the end of trial.

### Study outcomes

The primary outcome for the study was fasting plasma alpha-linolenic acid (ALA), which was measured at baseline and 6 months. Walnuts are one of the few dietary sources rich in ALA [30]. Prior studies have demonstrated that increased walnut intake leads to increased plasma ALA, suggesting that measurement of plasma ALA can be used as an objective biomarker to measure adherence to walnut supplementation [10, 31, 32]. Plasma ALA will be assessed using gas chromatography following previously published methods [33–35]. For quality control, identification, precision, and accuracy will be evaluated continuously with model mixtures of known fatty acid methyl esters and an in-house control pool, with identification confirmed by gas chromatography using a flame ionization detector. Based on prior experience, we expect high accuracy and precision for the measurement of ALA (with inter-assay coefficient of variation to be < 10%). Secondary outcomes included participants’ self-reported walnuts intake, adherence and acceptability of the supplement walnuts, as assessed by the 24-h diet recall and 7-item questionnaire. Exploratory outcomes included body weight, plasma lipids (total cholesterol, LDL-C, HDL-C, total triglycerides), and fasting blood glucose.

### Sample size

The sample size of 210 individuals (70 in each arm) provided more than 90% power with two-sided alpha of 0.05 to detect a mean difference of 0.12% (as percent of total fatty acid) in plasma ALA between the 30 g walnut supplementation and control arms [10]. The power estimation assumed a standard deviation (SD) of 0.18 and allowed for a drop-out rate of 10%. The study had similar power to detect a mean difference of 0.12% in plasma ALA comparing the 60 g/day and 30 g/day walnut supplementation groups. The power estimations were conservative since the assumptions were based on the PREDIMED trial that provided lower doses of walnuts (15 g/day) than the doses employed in this trial [10].

### Statistical analysis

Differences in between group post-treatment mean plasma ALA will be assessed by linear regression. In the primary analyses, study group assignment will be treated in the ANCOVA model as a categorical independent variable, with the control as the reference group. There will be three pair-wise comparisons: 1) 30 g/day walnut group vs. control, 2) 60 g/day walnut group vs. control, 3) 60 g/day walnut group vs. 30 g/day walnut group. Analyses will adjust for baseline plasma ALA and villages, and follow the intention-to-treat principle. An additional sensitivity analysis will be performed stratified by the baseline CVD status to examine any potential effect modification on the primary outcome.

For the secondary outcome of self-reported mean walnut intake (g/day) assessed by 24-h diet recalls, and the exploratory outcomes (plasma CVD lipids risk factors), differences in end of trial mean values between groups will be assessed by linear regression following the same approach as for the primary outcome.

Self-reported consumption patterns and satisfaction related to walnut intake will be assessed by the 7-item short questionnaire. The questionnaire included the following items: whether the participants consumed the walnuts, the ways they consumed the walnuts, and the number of times of walnuts intake in a day (each will be summarized as frequencies and %); as well as the number of days per week the participants consumed walnuts and their satisfaction rating on walnut flavour (will be summarized as mean ± SD).

Statistical significance is defined as two tailed α = 0.05.

### Data management

The trial data were centrally and securely stored on a Redcap data management server hosted by The George Institute for Global Health, China. Primary collection of screening, intervention, and follow-up visit data were done using the Redcap application on tablets. Quality of the data was ensured by pre-specified range and logic checks built within the Redcap system. All personal information about participants was de-identified and stored in a password protected secured computer.

### Participant characteristics

Participant recruitment started on October 16, 2019 and was completed on October 22, 2019. A total of 246 individuals were screened, and 210 met eligibility criteria and provided informed consent to participate in the trial. Ninety-four percent of participants were recruited on the basis of a history of CVD. Baseline characteristics of the participants were shown in Table 2. The participants were older adults (mean [SD] age = 65 ± 7 years) and approximately half were female (47%). Most participants attained up to primary school education (73%), with about 60% of participants were either past or current smokers. Only a small proportion were current drinkers (4.3%). Most participants had a history of ischemic heart disease (81%), and many also had a history of stroke (31%). Participants had similar baseline demographic and medical characteristics across the three randomized groups.

**Table 2:** Baseline characteristics of trial participants

	Total (*n* = 210)	Control (*n* = 70)	Low dose (*n* = 70)	High dose (*n* = 70)
Age (y), mean (SD)	65.4 (7.3)	64.4 (7.4)	64.7 (8.5)	67.0 (5.7)
Female (%)	46.7	47.1	50.0	42.9
Education				
Primary school or lower (%)	73.3	70.0	70.0	80.0
Junior high school (%)	18.1	17.1	18.6	18.6
Senior high school or above (%)	8.6	12.9	11.3	1.4
Past smoker (%)	44.3	44.3	40.0	48.6
Current smoker(%)	15.2	17.1	11.4	17.1
Past drinking (%)	34.3	35.7	34.3	32.9
Current drinking (%)	4.3	2.9	2.9	7.1
Body mass index (kg/m^2^), mean (SD)	26.4 (3.4)	26.5 (3.4)	25.8 (3.6)	26.3 (3.1)
Body weight (kg), mean (SD)	68.0 (11.1)	69.1 (11.6)	67.8 (12.5)	67.0 (9.0)
Waist hip ratio (SD)	0.94 (0.1)	0.96 (0.06)	0.94 (0.06)	0.93 (0.06)
Male (SD)	0.95 (0.1)	0.97 (0.07)	0.93 (0.06)	0.93 (0.06)
Female (SD)	0.93 (0.1)	0.93 (0.06)	0.94 (0.06)	0.93 (0.06)
Systolic blood pressure (mm Hg), mean (SD)	147.1 (19.9)	146.5 (19.9)	146.5 (21.8)	146.8 (18.1)
Diastolic blood pressure (mm Hg), mean (SD)	84.9 (12.0)	85.0 (12.9)	85.0 (12.3)	84.8 (10.9)
Medication use				
Lipid lowering agent (%)	67.1	55.7	78.6	67.1
Blood pressure lowering agent (%)	85.2	84.3	80.0	91.4
Disease history				
CVD^a^(%)	95.2	92.9	98.6	94.3
Stroke (%)	31.0	24.3	25.7	42.9
Transient ischemic attack (%)	29.5	21.4	21.4	45.7
Ischemic heart disease (%)	81.0	78.6	90.0	74.3
Congestive heart failure (%)	10.5	8.6	8.6	14.3
Peripheral arterial disease (%)	7.6	8.6	5.7	8.6
Hypertension (%)	83.3	81.4	80.0	88.6
Diabetes mellitus (%)	31.4	34.3	30.0	30.0

^a^Included stroke, transient ischemic attack, ischemic heart disease, congestive heart disease and peripheral arterial disease

## Discussion

The NUTS study successfully recruited 210 participants in less than 1 week, suggesting that the approach developed in this study can efficiently and rapidly recruit participants at high risk of CVD from rural villages in China to evaluate a walnut supplementation intervention. The evaluation of the acceptability and adherence to long-term walnut supplementation depended on the complete, unbiased follow up and outcome assessment of participants during the study period. The planned interim follow-ups aimed to engage and motivate participants to adhere to their assigned study group and should help to enhance the integrity of the trial.

The NUTS study was planned to support the design of a future large-scale, long-term, RCT in rural China that will test the effect of increased walnut intake on the risk of CVD. There is a growing recognition that nutrition science will benefit from more adequately-powered and well-conducted RCTs to evaluate the health effects of nutrients and foods on clinical outcomes, which can complement findings from prospective cohorts and metabolic studies [36]. These research approaches have different strengths and weaknesses, and consistency of findings can significantly enhance the development of dietary guidelines and their effective implementation. For instance, the seafood-derived, long-chain omega-3 polyunsaturated fatty acids have been shown to associate with lower risk of coronary heart disease (CHD) death in observational cohort studies, and reduce CHD deaths in several adequately powered RCTs [37, 38]. This coherent evidence base has important implications for current dietary and clinical guidelines to include omega-3 rich seafood for the prevention of coronary heart disease and omega-3 fatty acid supplement for the secondary prevention of CHD [9, 37, 39]. Our future planned RCT aims to similarly provide a critical part of the evidence base for nuts and cardiovascular health.

There were several notable strengths of the NUTS pilot study. First, this 3-arm randomized controlled trial allowed testing for a potential dose-response effect of walnut consumption by including two intervention groups. Second, the trial had sufficient power, and conservatively assumed an effect size based on that observed in the PREDIMED trial, which used a lower dose (15 g/day) of walnuts together with a mixture of 7.5 g/day of almonds and 7.5 g/day of hazelnuts [10]. Third, using plasma ALA as an objective biomarker to assess the primary outcome of adherence minimized the risk of measurement bias and errors associated with self-reported dietary intake. We also maintained blinding of all outcome assessors to further safeguard against ascertainment bias. Finally, the 6-month intervention duration was longer than the majority of previous trials, and provided evidence on the feasibility of nuts supplementation over an extended period [1, 2].

There were also limitations to this study. Firstly, participants were adults at high risk of CVD from rural Chinese villages, and findings of acceptability and adherence to walnut supplementation may not be generalizable to other populations. However, because the main purpose of the study was to aid the design and conduct of an RCT done in the same population, this was not a major concern. Secondly, this trial used a pragmatic approach with broad inclusion criteria that cover both individuals at high risk of CVD (without prior CVD) and those with previous CVD. These criteria were chosen to provide evidence about the feasibility of recruitment of participants for a future large trial (anticipated > 20,000) which would target both primary and secondary prevention. Such broad inclusion criteria are common for cardiovascular outcome trials [40, 41] where effects of interventions are typically homogenous across the groups. Increased nuts intake, for example, reduces LDL-cholesterol, which could be expected to contribute to both primary and secondary prevention [42, 43]. Finally, given the nature of the intervention, it was not possible to blind the participants to their randomized group, and there was a risk of bias as a consequence. To address this, outcome assessors were blinded to the group allocation and the primary outcome was an objective blood assay. It was possible that those in the control group may have altered their walnut intake if they became aware of the trial focus, and we evaluated this possibility using the dietary questionnaire surveys. However, sustained, daily increased intake of walnuts without provision appeared unlikely due to the relatively high price of walnuts compared to other commonly consumed foods in the area.

## Conclusions

In conclusion, the NUTS trial successfully recruited the targeted number of participants at high risk of CVD. The result of this trial will provide novel evidence to support the acceptability and adherence to long-term walnut supplementation in this population and will inform the design of a future CVD event-driven trial to test the effect of walnut supplementation for CVD prevention.

## Supplementary Information

**Additional file 1.**

## Data Availability

The datasets used and analysed during the current study are available from the corresponding author on reasonable request but restrictions apply to the availability of participants’ individual data under the Chinese data protection law.

## Abbreviations

CVD: Cardiovascular disease
ALA: Alpha linolenic acid
RCT: Randomized controlled trial
FFQ: Food frequency questionnaire
