# Supplementary material for: Protocol for a randomized controlled trial to test the acceptability and adherence to 6-months of walnut supplementation in Chinese adults at high risk of cardiovascular disease
Source: Nutr J. 2021 Jan 6;20:3. doi: 10.1186/s12937-020-00660-7 (PMC7789667; doi:10.1186/s12937-020-00660-7)
Supplement: Supplementary file 1 — Additional file 1. [file 12937_2020_660_MOESM1_ESM.zip › Supplementary material 7-item questionnaire end of trialR3.docx]

| 1. | Vital status | | | | | | | | | | | |
| --- | --- | --- | --- | --- | --- | --- | --- | --- | --- | --- | --- | --- |
| 1.1 | Follow-up date \|__\|__\|__\|__\| \|__\|__\| \|__\|__\| | | | | | | | | | | | |
|  | *yyyy* *mm dd* | | | | | | | | | | | |
| 1.2 | Yes | | No | | |  | | |  | | | |
|  | Y | | N | | | Completed this interview | | | | | | |
|  |  | |  | | | If no | | | Reasons of loss to follow up | | | |
| 1.2.1 |  | |  | | |  | | | Y | | Refuse | |
| 1.2.2 |  | |  | | |  | | | Y | | Cannot get contact | |
| 1.2.3 |  | |  | | |  | | | Y | | Participant died | |
| 1.2.4 |  | |  | | |  | | |  | | If yes | Death date: \|__\|__\|__\|__\| \|__\|__\| \|__\|__\| |
|  |  | |  | | |  | | |  | |  | *yyyy* *mm dd* |
| 1.2.5 |  | |  | | |  | | | Y | | Other, specify_________________________ | |
| 2. | Clinical assessment | | | | | | | | | | | |
| 2.1 | Height (cm) \|__\|__\|__\| | | | | | | | | | | | |
| 2.2 | Weight (kg) \|__\|__\|__\|.\|__\| | | | | | | | | | | | |
| 2.3 | Waist (cm) \|__\|__\|__\| | | | | | | | | | | | |
| 2.4 | Hip (cm) \|__\|__\|__\| | | | | | | | | | | | |
| 2.5 | Blood pressure Ⅰ \|__\|__\|__\|/\|__\|__\|__\| | | | | | | | | | | | |
|  | systolic diastolic | | | | | | | | | | | |
| 2.6 | Blood pressure Ⅱ \|__\|__\|__\|/\|__\|__\|__\| | | | | | | | | | | | |
|  | systolic diastolic | | | | | | | | | | | |
|  | Yes | | No | | |  | | |  | |  | |
| 2.7 | Y | | N | | | Blood sample taken | | | | | | |
|  | Yes | | No | | | D/K | | |  | | | |
| 3. | Y | | N | | | DK | | | Were you meant to eat NUTS study walnuts in the last week? | | | |
| 4. | How many days in the last week did you eat NUTS study walnuts? | | | | | | | | | | | |
|  | Y | | 1 | | |  | | |  | | | |
|  | Y | | 2 | | |  | | |  | | | |
|  | Y | | 3 | | |  | | |  | | | |
|  | Y | | 4 | | |  | | |  | | | |
|  | Y | | 5 | | |  | | |  | | | |
|  | Y | | 6 | | |  | | |  | | | |
|  | Y | | 7 | | |  | | |  | | | |
|  | DK | | Do not know | | | | | | | | | |
|  |  | |  | | | | | | | | | |
|  |  | |  | | | | | | | | | |
|  |  | |  | | | | | | | | | |
|  |  | |  | | | | | | | | | |
| 5. | If you were meant to eat NUTS study walnuts in the last week but did not eat them every day, why was that? | | | | | | | | | | | |
|  | Y | | Too hard to chew | | | | | | | | | |
|  | Y | | Did not like taste | | | | | | | | | |
|  | Y | | Ran out of nuts | | | | | | | | | |
|  | Y | | Other, specify | | | | | | | | | |
|  | DK | | Do not know | | | | | | | | | |
| 6. | If you ate NUTS study walnuts in the last week: | | | | | | | | | | | |
| 6.1 | How did you eat them? | | | | | | | | | | | |
|  | Y | | As a snack | | | | | | | | | |
|  | Y | | With meals | | | | | | | | | |
|  | Y | | Other, specify | | | | | | | | | |
|  | DK | | Do not know | | | | | | | | | |
| 6.2 | Did you consume them in one go or over two or more occasions during the day? | | | | | | | | | | | |
|  | Y | | One go | | | | | | | | | |
|  | Y | | Two or more occasions | | | | | | | | | |
|  | DK | | Do not know | | | | | | | | | |
| 6.3 | Did you enjoy the taste? (1 = disliked a lot; 10 = liked a lot) | | | | | | | | | | | |
|  | Y | | 1 | | | | | | | | | |
|  | Y | | 2 | | | | | | | | | |
|  | Y | | 3 | | | | | | | | | |
|  | Y | | 4 | | | | | | | | | |
|  | Y | | 5 | | | | | | | | | |
|  | Y | | 6 | | | | | | | | | |
|  | Y | | 7 | | | | | | | | | |
|  | Y | | 8 | | | | | | | | | |
|  | Y | | 9 | | | | | | | | | |
|  | Y | | 10 | | | | | | | | | |
| 7. | Overall over the last 6 months, about how many days did you eat the NUTS study walnuts? | | | | | | | | | | | |
|  | Y | | 24 (about 1 day a week) | | | | | | | | | |
|  | Y | | 48 (about 2 days a week) | | | | | | | | | |
|  | Y | | 72 (about 3 days a week) | | | | | | | | | |
|  | Y | | 96 (about 4 days a week) | | | | | | | | | |
|  | Y | | 120 (about 5 days a week) | | | | | | | | | |
|  | Y | | 144 (about 6 days a week) | | | | | | | | | |
|  | Y | | 168 (about everyday) | | | | | | | | | |
|  | DK | | Do not know | | | | | | | | | |
|  | Yes | | No | | | D/K | |  | | | | |
| 8. | Y | | N | | | DK | | If the walnuts were provided free would you consider continuing to eat them on a daily basis? | | | | |
|  |  | |  | | |  | |  | | | | |
|  | Yes | No | | |  | | |  | | | | |
| 9. | Y | N | | Have you had to stay in hospital for a night or more since the last follow up visit? | | | | | | | | |
|  |  | | If yes | | | | PLEASE FILL IN SAE FORM | | | | | |
|  |  | | | | | | | | | | | |
| 10. | Signature of the investigator | | | | | | | | | | | |
| 10.1 | Investigator name | | | | | | | | | \|__\|__\|__\|__\|__\|__\|__\|__\|__\|__\|__\|__\|__\|__\|__\|__\|__\|__\|__\|__\|__\|__\|__\|__\|__\|__\| | | |
| 10.2 | Investigator signature | | | | | | | | | \|____________________________________________________\| | | |
|  |  | |  | | | | | | | | | |
