# Supplementary material for: Protocol for a randomized controlled trial to test the acceptability and adherence to 6-months of walnut supplementation in Chinese adults at high risk of cardiovascular disease
Source: Nutr J. 2021 Jan 6;20:3. doi: 10.1186/s12937-020-00660-7 (PMC7789667; doi:10.1186/s12937-020-00660-7)
Supplement: Supplementary file 1 — Additional file 1. [file 12937_2020_660_MOESM1_ESM.zip › Supplementary material 7-item questionnaireR3.docx]

| 1. | Vital status | | | | | | | | | | |
| --- | --- | --- | --- | --- | --- | --- | --- | --- | --- | --- | --- |
| 1.1 | Follow-up date \|__\|__\|__\|__\| \|__\|__\| \|__\|__\| | | | | | | | | | | |
|  | *yyyy* *mm dd* | | | | | | | | | | |
| 1.2 | Yes | No |  | | | | |  | | |  |
|  | Y | N | Completed this interview | | | | | | | |  |
|  |  | | If no | | | Reasons of loss to follow up | | | | | |
| 1.2.1 |  | |  | | | Y | | | Refuse | | |
| 1.2.2 |  | |  | | | Y | | | Cannot get contact | | |
| 1.2.3 |  | |  | | | Y | | | Participant died | | |
| 1.2.4 |  | |  | | |  | | | If yes | Death date: \|__\|__\|__\|__\| \|__\|__\| \|__\|__\| | |
|  |  | |  | | |  | | | *yyyy* *mm dd* | | |
| 1.2.5 |  | |  | | | Y | | | Other, specify_________________________ | | |
|  | Yes | | No | | D/K | | | |  | | |
| 2. | Y | | N | | DK | | | | Were you meant to eat NUTS study walnuts in the last week? | | |
| 2.1 |  | |  | |  | | | | Why did not you eat the study walnuts in the last week? | | |
|  |  | |  | | Y | | | | Too hard to chew | | |
|  |  | |  | | Y | | | | Did not like taste | | |
|  |  | |  | | Y | | | | Ran out of nuts | | |
|  |  | |  | | Y | | | | Other, specify | | |
|  |  | |  | | DK | | | | Do not know | | |
| 3. | How many days in the last week did you eat NUTS study walnuts? | | | | | | | | | | |
|  | Y | | 1 | |  | | | |  | | |
|  | Y | | 2 | |  | | | |  | | |
|  | Y | | 3 | |  | | | |  | | |
|  | Y | | 4 | |  | | | |  | | |
|  | Y | | 5 | |  | | | |  | | |
|  | Y | | 6 | |  | | | |  | | |
|  | Y | | 7 | |  | | | |  | | |
|  | DK | | Do not know | | | | | | | | |
| 4. | If you were meant to eat NUTS study walnuts in the last week but did not eat them every day, why was that? | | | | | | | | | | |
|  | Y | | Too hard to chew | | | | | | | | |
|  | Y | | Did not like taste | | | | | | | | |
|  | Y | | Ran out of nuts | | | | | | | | |
|  | Y | | Other, specify | | | | | | | | |
|  | DK | | Do not know | | | | | | | | |
|  |  | |  | | | | | | | | |
|  |  | |  | | | | | | | | |
| 5. | If you ate NUTS study walnuts in the last week: | | | | | | | | | | |
| 5.1 | How did you eat them? | | | | | | | | | | |
|  | Y | | As a snack | | | | | | | | |
|  | Y | | With meals | | | | | | | | |
|  | Y | | Other, specify | | | | | | | | |
|  | DK | | Do not know | | | | | | | | |
| 5.2 | Did you consume them in one go or over two or more occasions during the day? | | | | | | | | | | |
|  | Y | | One go | | | | | | | | |
|  | Y | | Two or more occasions | | | | | | | | |
|  | DK | | Do not know | | | | | | | | |
| 5.3 | Did you enjoy the taste? (1 = disliked a lot; 10 = liked a lot) | | | | | | | | | | |
|  | Y | | 1 | | | | | | | | |
|  | Y | | 2 | | | | | | | | |
|  | Y | | 3 | | | | | | | | |
|  | Y | | 4 | | | | | | | | |
|  | Y | | 5 | | | | | | | | |
|  | Y | | 6 | | | | | | | | |
|  | Y | | 7 | | | | | | | | |
|  | Y | | 8 | | | | | | | | |
|  | Y | | 9 | | | | | | | | |
|  | Y | | 10 | | | | | | | | |
|  |  |  |  | | | | | | | |  |
|  | Yes | No |  | | | | | | | |  |
| 6. | Y | N | Have you had to stay in hospital for a night or more since you participated in this study? | | | | | | | |  |
|  |  | If yes | | PLEASE FILL IN SAE FORM | | | | | | | |
|  |  | | |  | | | | | | | |
| 8. | Signature of the investigator | | | | | | | | | | |
| 8.1 | Investigator name | | | | | | **\|**__\|__\|__\|__\|__\|__\|__\|__\|__\|__\|__\|__\|__\|__\|__\|__\|__\|__\|__\|__\|__**\|** | | | | |
| 8.2 | Investigator signature | | | | | | **\|**____________________________________________________**\|** | | | | |
